# SEATCA Tobacco Industry Interference Index: a tool for measuring implementation of WHO Framework Convention on Tobacco Control Article 5.3

**DOI:** 10.1136/tobaccocontrol-2014-051934

**Published:** 2015-04-23

**Authors:** Mary Assunta, E Ulysses Dorotheo

**Affiliations:** 1 Cancer Council Australia, Sydney, New South Wales, Australia; 2 Southeast Asia Tobacco Control Alliance, Manila, Philippines

**Keywords:** Tobacco industry, Denormalization, Public policy, Advocacy

## Abstract

**Objective:**

To measure the implementation of WHO Framework Convention on Tobacco Control (FCTC) Article 5.3 at country level using a new Tobacco Industry Interference Index and to report initial results using this index in seven Southeast Asian countries.

**Methods:**

Score sheet based on WHO FCTC Article 5.3 Guidelines sent to correspondents in seven Southeast Asian countries, using a scoring system designed with the help of tobacco control experts and validated through focused group discussions.

**Results:**

The seven countries ranked from the lowest level of interference to the highest are Brunei, Thailand, Lao PDR, Cambodia, Philippines, Malaysia and Indonesia. Countries that face high levels of unnecessary interaction with the tobacco industry also face high levels of tobacco industry influence in policy development. Most governments do not allow any tobacco industry representatives on their delegation to sessions of the Conference of the Parties or its subsidiary bodies nor accept their sponsorship for delegates, but most governments still accept or endorse offers of assistance from the tobacco industry in implementing tobacco control policies. Most governments also receive tobacco industry contributions (monetary or in kind) or endorse industry corporate social responsibility activities. Governments do not have a procedure for disclosing interactions with the tobacco industry, but Lao PDR, Philippines and Thailand have instituted measures to prevent or reduce industry interference.

**Conclusions:**

This Tobacco Industry Interference Index, based on the WHO FCTC Article 5.3 Guidelines, is a useful advocacy tool for identifying both progress and gaps in national efforts at implementing WHO FCTC Article 5.3.

## Introduction

Despite significant global progress in implementation of the WHO Framework Convention on Tobacco Control (FCTC)^1^ almost 10 years after it came into force, the degree of progress varies greatly across the treaty articles and across countries. A significant driver of this variance in implementation is the tobacco industry, which continues to actively interfere with FCTC implementation by seeking, directly or indirectly, to defeat, dilute and delay effective tobacco control measures.^2^ ^3^ Compliance with FCTC Article 5.3 to adequately address tobacco industry interference (TII) is therefore crucial in the effective implementation of the treaty. In order to provide more clarity and substance and to facilitate implementation, the Parties unanimously adopted the Article 5.3 Guidelines^4^ at the third Session of the Conference of the Parties (COP) in 2008 to encourage governments to establish, among other safeguards, measures to limit tobacco industry interactions with governments and to put in place public disclosure procedures.

Although Parties’ reports to the COP are informative, they convey little about the successes and challenges in FCTC implementation at the individual country level.^5^ In particular, information about Article 5.3 implementation is limited, and there has been no attempt so far to measure it systematically at country level. Recognising that TII is not easily recognised, understood or regularly monitored and countered by governments, despite the adoption of the Article 5.3 Guidelines, the Southeast Asia Tobacco Control Alliance (SEATCA) set out to develop a tool that would simplify the issue of TII and promote the use of the Article 5.3 Guidelines. The concept of a TII Index was born of discussions among Southeast Asian tobacco control leaders about how addressing TII is a matter of good governance and how Transparency International's Corruption Perceptions Index^6^ is both powerful yet easily understood.

This paper has two main aims: to measure the implementation of FCTC Article 5.3 at country level using a new Tobacco Industry Interference (TII) Index; and to report initial results using the index in seven Association of Southeast Asian Nations (ASEAN) countries: Brunei Darussalam, Cambodia, Indonesia, Lao PDR, Malaysia, the Philippines and Thailand.

## Methodology

The index is grounded in the Article 5.3 Guidelines, which make eight main recommendations with 34 subrecommendations to governments to ensure that the industry is prevented from exerting its influence on public health policy. In consultation with tobacco control experts from the region, 20 common TII incidents reported by SEATCA's tobacco control partners in the seven ASEAN countries were identified and referenced to 21 of the Article 5.3 Guidelines subrecommendations, before forming the basis for the 20 indicators in the TII Index. These 20 indicators, covering all eight main recommendations, were grouped into seven categories to help interpret the subrecommendations in a practical manner. A scoring system was developed depending on its applicability to each indicator. For most indicators, a sliding scale from one to five (never, rarely, sometimes, frequently or always) was used, but for those answerable by yes or no, scoring was limited to either 1 or 5. The indicators and scoring system are shown in table 1.

**Table 1:** Summary: tobacco industry interference index in ASEAN countries

Indicators	BN	KH	ID	LA	MY	PH	TH
**Level of participation in policy-development** *0 Not applicable, 1 Never, 2 Rarely (<10% of the time), 3 Sometimes (10–40% of the time), 4 Frequently (40–75% of the time), 5 Always (>75% of the time)*							
1. The government accepts, supports or endorses offer for assistance by or in collaboration with the tobacco industry in implementing tobacco control policies (Rec 3.1) *1 no incident, 2 receives/ accepts/ acknowledges, 3 supports or endorses, 4 uses assistance/repeats arguments, 5 allows such assistance or collaboration to influence decisions on policy	1	3	4	1	3	5	2
2. The government accepts, supports or endorses legislation drafted by/collaboration with the tobacco industry (Rec 3.4)	1	3	5	1	3	5	1
3. The government allows the tobacco industry to sit in multisectoral committee/advisory group that sets public health policy (Rec 4.8)	1	2	5	1	2	5	1
4. The government allows representatives from the tobacco industry (including state-owned) in the delegation to the COP or subsidiary bodies or accepts their sponsorship for delegates. (Rec 4.9 and 8.3)	1	1	0	1	1	5	1
Subtotal	4	9	14	4	9	20	5
**So-called CSR activities** *0 Not applicable, 1 Never, 2 Rarely (<10% of the time), 3 Sometimes (10–40% of the time), 4 Frequently (40–75% of the time), 5 Always (>75% of the time)*							
5. The government receives contributions from the tobacco industry (including so-called CSR contributions) (Rec 6.4)	1	3	5	2	2	4	4
6. The government agencies/officials endorses, forms partnerships with/ participates in tobacco industry CSR activities (Rec 6.2) *1 acknowledges, 2 endorses/supports, 3 participates (through officials), 4 forms partnership, 5 supports/partners with AND participates	0	5	5	0	2	3	4
Subtotal	1	8	10	2	4	7	8
**Benefits to the tobacco industry** *0 Not applicable, 1 Never, 2 Rarely (<10% of the time), 3 Sometimes (10–40% of the time), 4 Frequently (40–75% of the time), 5 Always (>75% of the time)*							
7. The government accommodates requests from the industry for longer implementation time or postponement of tobacco control law (Rec 7.1)	1	3	5	5	5	4	1
8. The government gives privileges, incentives, exemptions or benefits to the tobacco industry^i^ (Rec 7.3)	1	1	5	5	2	3	2
Subtotal	2	4	10	10	7	7	3
**Forms of unnecessary interaction** *1 Never 5 Yes (even if only 1 incident in the past 2 years)*							
9. Top-level government officials meet with/foster relations with the tobacco companies such as attending social functions and events sponsored or organised by the tobacco companies (Rec 2.1)	1	1	5	1	5	5	1
10. The government accepts assistance/offers of assistance from the tobacco industry on enforcement (Rec 3.1 and 4.3)	1	5	1	5	5	5	5
11. The government accepts, supports, endorses or enters into partnerships or agreements with the tobacco industry (Rec 3.1)	1	5	5	1	5	5	1
Subtotal	3	11	11	7	15	15	7
**Transparency** *0 Not applicable, 1 Never, 2 Rarely (<10% of the time), 3 Sometimes (10–40% of the time), 4 Frequently (40–75% of the time), 5 Always (>75% of the time)*							
12. The government does not publicly disclose meetings/interactions with the tobacco industry where such interactions are strictly necessary for regulation. (Rec 2.2)	0	5	5	3	5	5	1
Subtotal	0	5	5	3	5	5	1
**Conflict of Interest**							
13. The government does not have a policy (whether or not written) to prohibit contributions from the tobacco industry or any entity working to further its interests to political parties, candidates, or campaigns or to require full disclosure of such contributions (Rec 4.11) *1 No; 5 Yes	1	5	5	5	5	5	5
14. Retired senior officials work for the tobacco industry (Rec 4.4) *0 Not applicable, 1 Never, 2 Rarely (<10% of the time), 3 Sometimes (10–40% of the time), 4 Frequently (40–75% of the time), 5 Always (>75% of the time)	1	1	1	5	5	3	5
15. Current government officials and their relatives hold positions in the tobacco business including consultancy positions (Rec 4.5, 4.8 and 4.10) *0 Technical officials necessary to manage state-owned enterprise; 1 Low-to-mid-level public health officials; 2 Non-tobacco control high-level public health official, 3 Tobacco control-related official (agriculture, customs); 4 Tobacco control official in health ministry; 5 any high-level official (Minister, Prime Minister, including elected officials)	1	5	1	1	5	5	5
Subtotal	3	11	7	11	15	13	15
**Preventive Measures** *1 Yes, 2 Yes but partial only, 3 Policy/programme being developed, 4 Committed to develop such a policy/programme, 5 None*							
16. The government has a procedure for disclosing records of the interaction with tobacco industry and its representatives. (Rec 5.1)	5	5	5	5	5	1	2
17. The government has formulated, adopted or implemented a code of conduct for public officials, prescribing the standards they should comply when dealings with the tobacco industry (Rec 4.2)	5	4	5	5	3	1	1
18. The government requires the tobacco industry to periodically submit information on tobacco production, manufacture, market share, marketing expenditures, revenues and any other activity, including lobbying, philanthropy and political contributions. (Rec 5.2)	1	5	1	5	2	2	4
19. The government has a programme/system/plan to consistently raise awareness within its departments on policies relating to FCTC Article 5.3 Guidelines. (Rec 1.1, 1.2)	4	4	5	4	3	1	3
20. The government has a policy prohibiting the acceptance of all forms of contributions from the tobacco industry (monetary or otherwise) including offers of assistance, policy drafts or study visit invitations to the government, officials and their relatives. (Rec 3.4)	1	2	5	5	4	1	2
Subtotal	16	20	21	24	17	6	12
**Total**	**29**	**68**	**78**	**61**	**72**	**73**	**51**

^i^For example, The government reduced income tax rates or property tax exemption, duty-free imports of machineries and capital assets, subsidies for tobacco production, delayed implementation of excise tax increase, other incentives granted to foreign investors, duty-free tobacco distribution in government owned facility or shop.

ASEAN, Association of Southeast Asian Nations; COP, Conference of the Parties; CSR, corporate social responsibility; FCTC, Framework Convention on Tobacco Control.

In 2013 the score sheet was sent to civil society respondents in six ASEAN countries, who were established SEATCA partners, members of their national coalition, and thus knowledgeable about tobacco control. Respondents were asked to consult and, where necessary, have focused group discussions with advocates, partners and government and non-government organisations for purposes of filling up the score sheet. The index for Brunei Darussalam was completed by the government. The score sheet covered the period since the adoption of the Article 5.3 Guidelines (2009–2013). MA directly communicated with the respondents to ensure that each indicator was understood in the same way by all respondents and later reviewed all scores for purposes of verifying their accuracy. Respondents were required to provide publicly available evidence to support each score, although in some instances there may be no such evidence available other than personal knowledge or private communications.

Indicators under Conflict of Interest (Q 13–15) and Preventive Measures (Q 16–20) were scored based on a review or knowledge of existing policies related to tobacco control, particularly Article 5.3 and its Guidelines, to ascertain whether or not measures cited in the indicator exist. All other indicators were scored as a matter of perception supported by evidence. The scores are accurate as to the affirmation of incidents of interference, because evidence is provided for each score; however, scoring the intensity or scale of interference in a country is based on a collective assessment of the evidence available. Numeric equivalents of ‘rarely, frequently, and always’ (eg, >75% of the time) are not intended to be a quantification of the evidence available; rather, such scores represent the collective perception of civil society tobacco control advocates in a country.

Similarly, a country's score that is twice another's does not necessarily mean that it has twice as much TII. Thus to ensure that each country score was appropriate and reflected real-life perceptions, including comparisons with other countries, after individual country scores were entered into a regional score sheet, the results were reviewed collectively by all the respondents. A third party consultant also reviewed the results to verify accuracy of answers from a regional perspective.

## Findings

Preliminary findings were reported in mid-2013, and a final regional report was published in January 2014.^7^ The overall scores serve as a gauge of the level of TII based primarily on evidence available publicly as well as stakeholders’ perception. Among the 7 countries Brunei had the lowest level of interference, while Indonesia had the highest (see table 1).

### Tobacco industry participation in policy development

**Figure 1:**
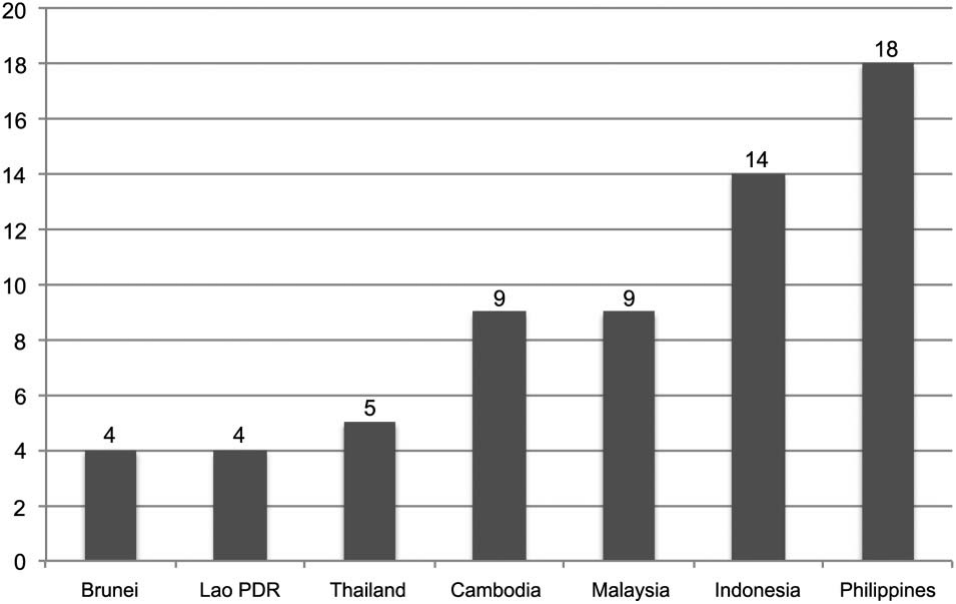
Tobacco industry participation in policy development.

Brunei, Thailand and Lao PDR curb industry participation, while the Philippines and Indonesia have high levels of participation (figure 1). On the positive side, most of the governments do not allow representatives from the tobacco industry in their delegations to the COP sessions or other FCTC-related meetings nor accept any industry sponsorship to attend these meetings.

Brunei does not allow the tobacco industry to participate in health policy development, accepts no contributions from the tobacco industry or corporate social responsibility (CSR) activities, gives no benefits to the industry, has no unnecessary interaction with the industry and requires the industry's representatives to provide information periodically. It must be noted that Brunei does not grow tobacco, has no cigarette manufacturing facilities and has a small tobacco market. Lao PDR, Malaysia and Thailand also do not allow the tobacco industry a seat in the multisectoral committee/advisory group that sets public health policy. The Philippines’ Tobacco Regulation Act (RA9211) gives the tobacco industry a seat in the Inter-Agency Committee on Tobacco (IACT).^8^ This legislation was enacted in 2003 before the Philippines ratified the FCTC and can only be amended by its Congress. The governments of Indonesia and Malaysia accept, endorse or consider legislation drafted by or in collaboration with the tobacco industry. Since Indonesia is a non-Party to the FCTC and does not attend FCTC meetings, it did not score on the issue of industry representation in delegations.

### Industry-related CSR activities

Tobacco companies have in recent years increased their CSR spending in the ASEAN region. Philip Morris International (PMI), for example, increased its charitable spending in 6 countries in the ASEAN region from US$8.2 million in 2009^9^ to US$10.2 million in 2012.^10^ In the Philippines and Thailand, PMI more than doubled its spending, while in Malaysia it increased its CSR expenses by fivefold. Despite the FCTC Article 13 Guidelines, CSR activities by the tobacco industry are not banned among ASEAN countries, although a few (Cambodia,^11^ Singapore^12^ and Thailand^13^) have banned their publicity. This presents a loophole, which the industry exploits. An exposé from the Philippines^14^ shows how tobacco companies use CSR activities to circumvent laws regulating the industry and as a strategy to gain access to elected officials with the power to approve and implement tobacco control policies. All governments, except Brunei, receive some form of contributions (monetary or otherwise) from the tobacco industry, and with the exception of Brunei and Lao PDR, government agencies or officials endorse tobacco industry CSR activities or form partnerships with the industry in receiving contributions.

### Benefits to the tobacco industry

Except for Brunei and Thailand, other governments provide benefits and privileges to the tobacco industry, such as accommodating requests for a longer time for or a postponement of tobacco control law implementation, unrelated to any lawsuits. For example, Malaysia's implementation of a ban on kiddie packs (less than 20 sticks) enacted in 2004^15^ was deferred for 6 years till May 2010 by a cabinet directive to help the industry.^16^ Also in Malaysia, restaurants and eating places are not 100% smoke-free^17^ to accommodate requests from the tobacco industry. Similarly, Indonesia granted the industry 18 months to apply pictorial health warnings (PHW) on cigarette packs effective mid-2014,^18^ although Indonesian tobacco companies have long been exporting packs with PHW to Brunei, Malaysia and Singapore. This is consistent with Indonesia's 14-year (2007–2020) Tobacco Roadmap, which favours the industry by prioritising labour and revenues before health.^19^ Indonesia's complicated multi-tiered tobacco tax structure also protects and provides privileges to the industry, while in Lao PDR the industry obtained an initial 5-year tax holiday and capped ad valorem tobacco tax rates at between 15% and 30% for 25 years (till 2026).^20^

When it is reported no incentives were accorded to the tobacco industry, it does not mean that absolutely no benefit was given. The presence of foreign tobacco investors in a country would indicate they naturally enjoy foreign investor privileges, but information on this may not be publicly available to advocates.

### Forms of unnecessary interaction

Overall Cambodia, Indonesia, Malaysia and the Philippines report high levels of interactions with the tobacco industry that are unnecessary for its regulation, supervision or control. These four countries also face high levels of tobacco industry participation in policy development. Cambodia, Indonesia, Malaysia and Thailand accept assistance or offers of assistance from the tobacco industry on enforcement such as conducting raids on tobacco smuggling or enforcing smoke-free policies. For example the Royal Malaysian Customs collaborated with the Confederation of Malaysian Tobacco Manufacturers (CMTM) in conducting antismuggling activities in 2010^21^ and continues this cooperative partnership in tackling the illicit cigarette problem in Malaysia.

Fortunately, Brunei, Cambodia, Lao PDR and Thailand report that top-level government officials do not meet with nor foster relations with tobacco companies, such as by attending social functions and events sponsored or organised by the tobacco companies. In 2009 when *Tabinfo*, a tobacco industry trade event, was held in Bangkok, although Thailand has a state tobacco monopoly, government officials were instructed not to endorse the event.^22^

### Transparency

Except in Thailand, the perception of respondents is that most governments do not publicly disclose meetings or their interactions with the tobacco industry or have not put in place procedures that enable them to do so. This includes not indicating when meetings with the industry take place, their purposes or the contents and outcomes of the meetings. The public is often informed of government decisions after such meetings through press statements. In the Philippines in 2010, the Civil Service Commission (CSC) and Department of Health (DOH) issued Joint Memorandum Circular (JMC) 2010–01 to protect the government bureaucracy against tobacco industry interference in accordance with the FCTC Article 5.3 Guidelines,^23^ and full implementation of the JMC for all its provisions and in all government agencies is still ongoing. Although the JMC requires transparency of necessary interactions, many government agencies still do not routinely disclose such interactions.

### Conflict of interest

Brunei prohibits political contributions from the tobacco industry. Other countries neither prohibit nor require full disclosure of such contributions from the tobacco industry or any entity working to further its interests to political parties, candidates or campaigns. In Malaysia and Thailand, current and retired senior officials and their relatives have worked for the tobacco industry. In Malaysia, for example, the former Attorney-General (1980–1993) on his retirement became the Chairman of BAT Malaysia (1994–2012).^24^ In Thailand in 2013 the Permanent Secretary of the Ministry of Interior was on the Thai Tobacco Monopoly's executive board,^25^ while a Minister in the Prime Minister's Office owns a tobacco leaf business.^26^ Overall there was limited information on this in countries; responses were subject to advocates’ own knowledge, although respondents who answered positively provided evidence. It is also possible that those who replied ‘never’ do not have the information or simply do not have the perception that this is an issue.

### Preventive measures

**Figure 2:**
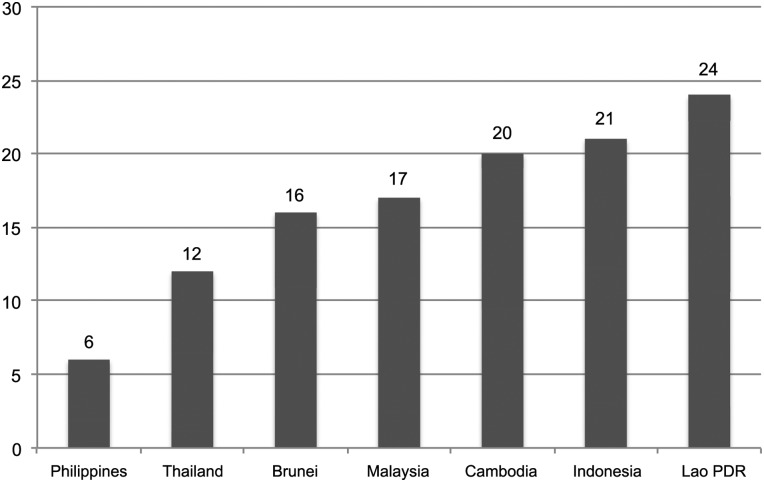
Measures to prevent tobacco industry interference.

Most governments do not have a procedure for disclosing records of interactions with the tobacco industry and its representatives. Philippines and Thailand are doing better than other countries in instituting measures to restrict tobacco industry interaction (figure 2). As recommended in the FCTC Article 5.3 Guidelines, one way to de-normalise the tobacco industry is through a code of conduct for officials when dealing with the tobacco industry. The Philippines’ CSC-DOH JMC 2010–01,^20^ which limits government interactions with the industry and rejects partnerships with tobacco companies, is the first of its kind in Asia, a result of the CSC's commitment to protect and promote the health of government workers and its realisation of the need to protect the whole of government, particularly non-health agencies, from the threat of TII in FCTC implementation. Thailand and Lao PDR have a similar policy for their respective ministries of health only.

Through a cabinet decision, Thailand prohibits the acceptance of all forms of contributions from the Thai Tobacco Monopoly, including offers of assistance, policy drafts or study visit invitations to the government and its officials; however, this does not apply to other tobacco companies. Brunei, Indonesia, Malaysia and Thailand require the tobacco industry to submit information on tobacco production, manufacture, market share, and revenues; however, the tobacco industry is not required to provide information on marketing expenditures, expenses on lobbying or philanthropic and political contributions.

## Discussion

### Using the TII index

The TII Index was designed to systematically measure FCTC Article 5.3 implementation at country level. As far as we know, this has never been carried out before, although scoring for other tobacco control interventions has been previously described.^27^ Using the index, it is hoped that governments and advocates can identify their countries’ challenges and begin to address them.

Overall countries still have a long way to go in implementing Article 5.3. While governments have made efforts to raise awareness on TII and policies relating to Article 5.3, these are not carried out in a systematic and consistent manner. This is why no country has a perfect score and why efforts to prevent and reduce TII are in a constant state of flux. For example, Malaysia, which has fairly stringent tobacco control measures, shows strong industry influence, possibly explaining why Malaysia still does not have 100% smoke-free restaurants and workplaces. Similarly, regardless of its being a non-Party to the FCTC, Indonesia's poor performance in this index shows the many areas influenced by the industry, which helps explain why Indonesia is lagging in tobacco control behind other countries in the region.

Poor or imperfect scores should not discourage governments but rather should be a starting point for discussing what is achievable in the country context. The Philippines, for example, has performed well in putting in place preventive measures through the JMC; however, the index clearly shows that the industry can interfere and influence policy through the IACT and through its increased CSR spending. Hence, in addition to fully implementing the JMC across the whole of government, the next step for the Philippines to consider will be to amend its legislation to exclude the tobacco industry from the IACT and ban CSR activities by the industry. Countries will recall that the Philippine tobacco industry was previously described as the ‘strongest tobacco lobby in Asia’^28^ and may wish to follow the lead of the Philippines’ JMC; although given the various political realities in each country, there is likely no one-size-fits-all solution to preventing TII. Prioritisation also needs to be carried out within each country's context.

Based on the index findings, however, the following are some practical considerations.

- Since FCTC implementation requires a ‘whole of government’ approach and not just actions by the Ministries of Health, it is vital that relevant non-health actors increasingly be made aware of TII within their sectors, as well as their roles in implementing the Article 5.3 Guidelines. This can lead to the establishment of a multisectoral government body that will take charge of coordinating industry monitoring (industry lobbying, unnecessary interactions, benefits to the industry and industry CSR) and responses to identified TII.
- Most governments already have codes of conduct for civil servants. A code of conduct based on the Article 5.3 Guidelines can be developed (or existing ones amended) to require increased transparency and thorough disclosures of interactions with and benefits given to the tobacco industry and to prohibit partnerships with or contributions from the industry.
- Banning so-called CSR activities by the tobacco industry and requiring the tobacco industry to disclose periodically information (on tobacco production, manufacture, market share, revenues, expenditure on marketing, lobbying, philanthropy and political contributions) can be included in the code of conduct or carried out through legislation.

If such efforts are systematically stepped up in all countries, these will assist governments towards better compliance with the FCTC in general and the Article 5.3 Guidelines in particular.

A thorny situation arises when a government has a state-owned tobacco business where the tobacco industry can double-up as a government department and the industry, even if the Article 5.3 Guidelines state clearly to treat state-owned tobacco industry in the same way as any other tobacco industry (Recommendation 8). Although it has a state-owned tobacco enterprise, Thailand serves as a good example of a country that has performed well in tobacco control compared to other countries.

### Limitations

Respondents were limited by the information available to them publicly, through the Internet, newspapers, government records and tobacco company reports; hence the results for most indicators tend to represent a conservative view. This is more pronounced in countries that provide limited access to government information. Thus scores that imply that unnecessary interactions had not happened or incentives had not been given since 2009 do not necessarily mean these do not exist for some countries. In countries where there are no policies, written or not, to prohibit or limit unnecessary interactions and where access to public information is low (such as Cambodia, Laos and Indonesia), it is possible that interactions occurred, but the respondents had no public evidence to point to. Further research is needed to better corroborate this information.

Since TII comes in many forms, it was not possible to agree on a weighting system that could be applied uniformly across countries. In addition, scores equivalent to ‘rarely, frequently, and always’ are a subjective determination; however, it is hoped that the collective perception of civil society tobacco control advocates in a country provides a sufficient level of objectiveness to the index scores.

## Conclusion

In order to accelerate FCTC implementation, governments and civil society need to be more proactive in de-normalising the tobacco industry.^29^ Examining the index results, learning from the successes and mistakes of other countries and anticipating the challenges ahead can facilitate this seemingly daunting task. We hope the index will be used in other regions, encouraging comparisons and sharing of experiences between countries and thus increasing the motivation to continually reduce TII.

We also acknowledge that this index is a work in progress. Its initial application in seven countries is only a first step in examining Article 5.3 implementation. If it is applied to other regions, we would like to know if the 20 indicators are equally valid in those other regions, if they need to be expanded to encompass all the subrecommendations in the Article 5.3 Guidelines, and how the scoring might be further improved.

**What this paper adds**

- This is the first ever index developed to score and rank countries in their implementation of WHO Framework Convention on Tobacco Control (FCTC) Article 5.3 Guidelines. Countries face many challenges in implementing Article 5.3 and this Index simplifies the various categories of industry interference and aims to show where the areas of weakness lie. The Index also recognises countries that have taken measures to strengthen the bureaucracy and protect themselves from the tobacco industry.
